# Antiviral Strategies for Pandemic and Seasonal Influenza

**DOI:** 10.3390/v2081766

**Published:** 2010-08-20

**Authors:** Maria Hedlund, Jeffrey L. Larson, Fang Fang

**Affiliations:** NexBio, Inc./10665 Sorrento Valley Rd, San Diego, CA 92121, USA; E-Mails: mhedlund@nexbio.com (M.H.); jlarson@nexbio.com (J.L.L.)

**Keywords:** influenza virus, neuraminidase inhibitors, sialic acid, RNA polymerase

## Abstract

While vaccines are the primary public health response to seasonal and pandemic flu, short of a universal vaccine there are inherent limitations to this approach. Antiviral drugs provide valuable alternative options for treatment and prophylaxis of influenza. Here, we will review drugs and drug candidates against influenza with an emphasis on the recent progress of a host-targeting entry-blocker drug candidate, DAS181, a sialidase fusion protein.

## 1. Introduction

Infectious outbreaks with influenza virus are associated with high disease-related mortality and significant socioeconomic impact. In the United States alone, annual epidemics cause approximately 300,000 hospitalizations and 36,000 deaths. In addition, three influenza pandemics (1918, 1957 and 1968) during the recent century have together taken an enormous toll of millions of lives and cost billions of dollars. The appearances of the avian H5N1 influenza virus in 2003, and the more recent pandemic H1N1 outbreak in 2009, serve as stark reminders that preparedness to meet the threat of new and infectious influenza virus is essential. Although vaccines remain the primary public health response to a potential pandemic outbreak, shortages in vaccine production and the time required to deliver a vaccine against a novel influenza virus strain present considerable challenges to public protection against influenza virus. To cover this gap in vaccine production, and to treat individuals already infected, stockpiling of antiviral drugs is becoming commonplace.

Two classes of antiviral compounds are currently on the market, the M2 inhibitors such as amantadine and rimantadine; and the viral neuraminidase inhibitors (NAIs) oseltamivir (Tamiflu®; GlaxoSmithKline) and zanamivir (Relenza®; Roche). The former class is effective primarily for influenza A, while the NAIs, which entered the market place in recent years, work for both influenza A and B viruses. The M2 inhibitors are also commonly associated with side effects and their effectiveness is hindered by wide-spread drug resistance. The rate of M2 resistance started to increase in 2003, primarily in H3N2 strains, reaching greater than 90% in 2006. The H1N1 strains did not develop M2 resistance to the same extent. However, due to the high rate of resistance, in 2006 physicians were advised to stop prescribing rimantadine and amantadine [1]. With the increased use of the NAIs, more and more influenza virus strains are found to develop resistance [1,2], and in 2008, the CDC reported that close to 100% of seasonal H1N1 isolates had become oseltamivir resistant [3]. While this is a problem during any seasonal outbreak, this might pose a greater problem if a resistance gene was re-assorted into a potential pandemic strain, especially in light of the recent H1N1 pandemic and the high-rate of oseltamivir resistance in circulating seasonal strains. Similar to previous pandemic influenza virus, the 2009 pandemic H1N1 virus was the result of re-assortments of influenza virus from different species (swine, avian and human) [4].

Influenza viruses are members of the Orthomyxoviridae family and are enveloped RNA viruses with eight segmented negative-strand RNA genomes. The genome is enclosed by the viral envelope which is decorated with three different proteins; the hemagglutinin (HA), neuraminidase (NA), and M2 ion channel. The HA attaches virus to host cell receptors and mediates fusion of viral and cellular membranes. The NA facilitates release of new viruses from the host cell; and a small number of M2 proteins ion channels mediate viral uncoating. The currently approved antiviral drugs target the M2 protein and the NA. Further insight into the mechanisms of influenza virus infection has led to the development of novel therapeutic candidates designed to target various steps of the virus life cycle including host cell adhesion, viral RNA replication, and, release of virions from infected cells. This review will summarize the state of current therapeutics along with new drugs currently in clinical development.

## 2. Currently available therapeutics

**Figure 1.**
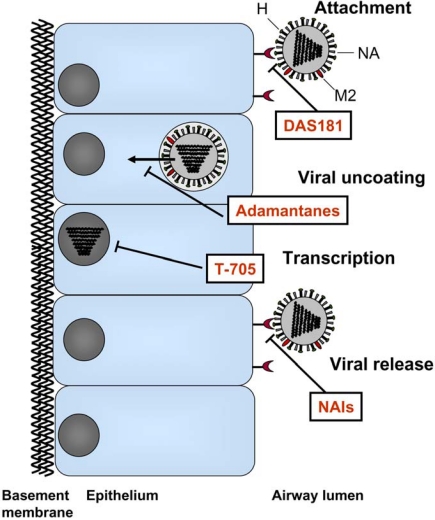
Targets for current therapeutics and new drugs in clinical development.

Traditional antivirals have been pathogen-targeted (Figure 1). For example, the first class of antiviral drugs developed for the treatment of influenza virus was the adamantanes which block the viral M2 ion channels. The first M2 blocker amantadine was introduced to the market in 1967 followed by the second M2 blocker, rimantadine, 26 years later. These drugs block the M2 ion channels which mediate the uncoating of the viral particle inside the host cell. Due to the increasing rate of resistance to the adamantanes and the inherent toxicity of these drugs, the predominant type of antiviral drug currently used for the treatment and prophylaxis of influenza are NA inhibitors (NAIs). These drugs are sialic acid analogues which bind to the viral NA and thereby interfere with the release of new influenza virus particles from infected cells.

### 2.1. M2 ion channel blockers

Clinical studies have shown that amantadine and rimantadine prophylaxis resulted in a reduction of 60–70% of the total number of influenza virus A cases, and shortened the duration of illness by 1 day in adults [5]. An obvious problem with amantadine and to a lesser extent rimantadine, are the many neuropsychiatric side effects such as insomnia, confusion, hallucinations, ataxia, difficulty in concentration, depression, dizziness and tremor; as well as gastrointestinal side effects including anorexia, nausea and vomiting [5]. Currently adamantanes are limited in their clinical use for influenza virus because of widespread drug resistance. Amantidine-resistant influenza A virus was first detected in the early eighties and had by 2005 reached 90% [6]. The increase in resistance does not appear to be dependent on the increase in usage, since resistance was found prevalent in countries with low prescription rates [6]. The majority of the resistance results from Ser31–Asn mutation in the M2 gene and to a lesser extent position 27 or 30. Resistance develops readily after three or five passages in cell culture, and about 30% of patients shed drug resistant virus. The 2009 H1N1 was found to be resistant to both amantadine and rimantadine [7–9].

### 2.2. NAIs

The NAIs currently on the market were approved in 1999 and are now the predominant class of antivirals used for the treatment of existing cases of influenza virus and prophylaxis of potential outbreaks. Indeed, stockpiling of NAIs is standard practice in many countries in response to the growing threat of pandemic influenza virus. The NAIs interfere with the release of new influenza virus particles from infected cells, and hence prevent the infection of neighboring cells or transmission to uninfected individuals. In general, this class of antivirals is associated with reduced toxicity compared to the adamantanes, and unlike the adamantanes, the NAIs are effective against both influenza A and B. Post-licensing reports have shown that zanamivir may cause cough, bronchospasm or even death in patients with pre-existing pulmonary disease and therefore this antiviral is contraindicated for patients with serious underlying respiratory diseases [10]. Oseltamivir have been shown to cause transient nausea, vomiting and abdominal pain in up to 10% of patients [11]. Additionally there have been some cases of delirium and behavioral problems following the use of NAIs [11]. Meta-analyses of the treatment effectiveness of NAIs, demonstrated that zanamivir and oseltamivir effectively reduced the period of time for influenza symptoms by 0.8 and 0.9 days respectively in adults [12]. Furthermore, they were shown to decrease the risk of complications from influenza [5]. Zanamivir pre-/post- prophylaxis studies of against seasonal influenza virus have shown a 69–81% reduction in incidence compared to placebo. For oseltamivir, the same numbers were 74% and 90% pre-/post-exposure respectively. While NAIs provided no clear benefit when treatment was initiated beyond 48 h after onset of symptoms in previously healthy adults, it is not yet known if immune compromised individuals may benefit from treatment initiated at later time-points. [13]

The NAIs are sialic acid receptor analogues, so any mutation within the NA molecule was initially thought to decrease the actual fitness of the virus [2,14]; however, with 99% of seasonal H1N1 isolates being oseltamivir resistant it is evident that the virulence is not significantly impaired [3]. The most common mutation associated with oseltamivir resistance is the H274Y (histidine to tyrosine at codon 274 in N2 nomenclature or H275Y in N1 nomenclature). Although almost 100% of seasonal H1N1 influenza virus tested in 2009 were reported to be resistant to oseltamivir[15], the majority of the 2009 H1N1 pandemic isolates tested were found to still be susceptible to the NAIs. However rare cases and primarily immuno-compromised individuals were found to be infected with isolates of the 2009 pandemic H1N1 that were carrying the H274Y mutation [16–20]. Zanamivir serves as the only alternative antiviral drug for treatment of oseltamivir resistant influenza [14,21]. Noticeably, certain 2009 influenza virus clinical isolates have been reported to be resistant to both oseltamivir and zanamivir *in vitro* [22], although the clinical significance of the finding remains unclear. Zanamivir resistance has also been observed in isolates from zanamivir-treated immuno-compromised individuals. [23,24]

## 3. New Antiviral Agents for Influenza Treatment

The common goal for the development of new antiviral agents is to create drugs that provide more effective and more broad-spectrum influenza treatment compared to current treatments, as well as to reduce the potential for drug resistance. Potential approaches to develop new treatments for influenza were previously reviewed [25–28]. The antiviral agents that are now in various clinical stages of development which will be discussed here (Figure 1) are listed in Table 1.

## 4. Pathogen-targeted approaches

### 4.1. NA inhibitors

Parenteral NAIs. In order to promote nearly-instant delivery of therapeutically relevant doses to sites of infection, especially in cases of extrapulmonary infection, parenteral administration of currently approved drugs may provide a useful alternative to oral or inhalation delivery. Due to low bioavailability (2%) zanamivir is not amenable to the oral route of delivery [29], however the intravenous route of delivery of zanamivir is currently being evaluated in phase 2a clinical trials [30]. The intravenous route was found to be highly effective in healthy individuals receiving prophylaxis of 600 mg zanamivir or placebo twice daily for 5 days prior infection; zanamivir reduced the infection rate by 76% and to prevented illness [30]. Furthermore, intravenous zanamivir was shown to protect against H5N1 influenza virus in a primate model [31], and clinical testing against avian influenza A(H5N1) is underway in China [32].

Peramivir (BioCryst) is an NAI under development for the treatment and prevention of influenza virus. Similar to oseltamivir and zanamivir, peramivir is a potent inhibitor of influenza virus NA, with *in vitro* IC50 data in the low nanomolar range [33]. Peramivir has a prolonged plasma half-life in humans and also appears to bind to the enzyme for a prolonged time, which could allow for less frequent dosing [34]. Similar to zanamivir, peramivir has shown to have low oral bioavailability [35], and is further developed for intravenous delivery. In dose-range finding studies, intravenous delivery resulted in a 2-fold increase in peak plasma levels compared to oseltamivir following oral delivery [36]. While, two separate studies using intramuscular delivery of peramivir failed to show any decrease in time to alleviation of symptoms [37,38], intravenous delivery significantly improved the time to reach clinical stability in hospitalized patients [39]. Phase 3 data for intravenous peramivir showed no benefit relative to oseltamivir against acute influenza. Furthermore, there appears to be some cross-resistance between strains carrying the H274Y mutation which may limit the use of peramivir [40,41].

CS-8958 (laninamivir; Biota) is a long-acting NAI which is an esterified form of a molecule structurally similar to zanamivir [42]. CS-8958 is being co-developed by Biota Holdings of Australia and Daiichi-Sankyo Pharmaceuticals of Japan. A phase 3 clinical trial demonstrated that a single dose of CS-8958 was comparable to a standard treatment with oseltamivir [43]. No published data are available regarding resistance to CS-8958.

### 4.2. Polymerase inhibitors

T-705 (favipiravir) (6-fluoro-3-hydroxy-2-pyrazinecarboxamide) developed by Toyama Chemical Co. Ltd. (a subsidiary of Fuji Holdings), is a non-peptide small molecule candidate influenza drug that targets the viral gene replication process. T-705 prevents viral reproduction through inhibition of the viral RNA polymerase [44] similar to the action of ribavirin. T-705 appears to be more specific than other RNA polymerase inhibitors, and is thought to have reduced toxicity compared to ribavirin [45,46]. T-705 has shown selective inhibition of activity of a wide array of influenza A, B and C viruses *in vitro*, as well as *in vitro* and *in vivo* activity against H5N1, pandemic H1N1 strains, members of the arenavirus family, West Nile virus and yellow fever virus [47–51]. T-705 is currently in Phase II clinical studies for the treatment of influenza.

## 5. Host-targeted approaches

At present, the only host-targeting drug candidate under clinical development is DAS181 (Fludase^®^; NexBio, Inc.), a 46 kDa recombinant fusion protein consisting of a sialidase catalytic domain from *Actinomyces viscosus*, a common oral bacterium, linked to a human amphiregulin glycosaminoglycan epithelium-anchoring domain [52]. The anchoring domain mediates retention of the drug on the airway mucosa at the site of action. By cleaving sialic acids from the host cell surface, DAS181 inactivates the host cell receptors for influenza and parainfluenza virus [53], thereby preventing the attachment of virus and consequently blocking viral entry into respiratory epithelial cells. (Figure 1).

DAS181 cleaves both the α2,6-sialic acid–linked receptors to which human viruses attach and the α2,3-sialic acid–linked receptors to which avian viruses attach, and has been shown inhibitory for a large number of influenza A and B viruses, with *in vitro* EC90 values in the low nanomolar range [52]. The epithelial tag on this molecule increases its activity by an order of magnitude (∼5–30-fold) [52]. DAS181 has been shown to potently inhibit influenza and parainfluenza virus replication in various *in vitro* and *ex vivo* cell and tissue culture model systems, including various cell lines, well-differentiated human airway epithelium cultures (HAE), primary human type I pneumocytes, human lung biopsy tissue, and human bronchial tissue sections. The *in vitro* antiviral activity of DAS181 has been demonstrated against over 60 laboratory strains and clinical isolates of influenza virus A of the H1N1, H2N2, H3N2, H5N1, H9N2, and H7N7 subtypes, multiple influenza virus B strains, and all three subtypes of human parainfluenza virus (PIV-1, -2, and -3). The *in vivo* efficacy of DAS181 and its analogue has also been demonstrated in mouse, ferret, and cotton rat against different influenza A virus subtypes (seasonal H1N1, Pandemic H1N1, H3N2, and H5N1), influenza virus B, and parainfluenza virus type 3. Significant efficacy of DAS181 has also been demonstrated when treatment was initiated either prophylactically or at up to 3 days post-infection. These results have been published [52,54–57].

With its broad-spectrum activity DAS181 is active against all seasonal influenza strains tested, including clinical isolates carrying the H274Y mutation [22,58], as well as highly pathogenic avian influenza strains (H5N1). [52]. The potent activity of DAS181 against clinical isolates of Pandemic 2009 influenza A virus (H1N1) as well as circulating NAI-resistant influenza virus strains has been demonstrated *in vitro, ex vivo* and *in vivo* [22,59]. A similar level of potency of DAS181 was observed against all influenza virus clinical isolates from the recent years, including the pandemic strains as well as the ones with marked resistance to NAIs [22,59]. These findings highlight the potential broad spectrum activity of DAS181 against novel and drug-resistant influenza virus strains.

The potential for influenza virus to develop resistance to DAS181 has been evaluated by subjecting influenza virus strains to increasing selective pressure under DAS181 over multiple *in vitro* passages (up to 34 passages). The DAS181 selected virus strains exhibited <20-fold resistance to DAS181. In contrast to the low level resistance to DAS181, under similar *in vitro* selection conditions, influenza virus developed >1000 fold to >100,000 fold resistance to NAIs within less than 15 passages [60–70]. DAS181-selected virus exhibited an attenuated phenotype *in vitro* and *in vivo*. In comparison to wild type counterparts, DAS181-selected virus produced 4–6 fold smaller plaques, was less virulent in mice, and retained sensitivity to DAS181 *in vivo* in mice at standard therapeutic doses. Importantly, the DAS181-resistant phenotype appears to be unstable as DAS181-selected strains quickly regain sensitivity to DAS181 after withdrawal of DAS181 from the viral cultures (in manuscript). By contrast, the NAI-resistant influenza virus strains are phenotypically and genotypically stable [71]; reversion to NAI sensitivity upon NAI withdrawal has not been reported to our knowledge. These results suggest a markedly reduced potential for selecting resistance to DAS181.

Secondary bacterial infection following influenza virus infection is a major health concern due to the increased risk for mortality. Pre-clinical studies have demonstrated that DAS181 treatment did not increase colonization of respiratory epithelial cells by *Streptococcus pneumoniae*, *Haemophilus influenzae* or *Pseudomonas aeruginosa* [55]. In healthy mice, DAS181 treatment did not increase *S. pneumoniae* colonization in the respiratory tract [72]. In influenza virus infected mice, DAS181 prevented bacterial pneumonia and mortality, inhibited influenza virus titer by ≥3 logs, and significantly decreased bacterial colony counts compared with untreated animals. None of the treated animals showed enhanced *S. pneumoniae* lung colonization. Additionally, DAS181-treated mice were free from opportunistic infections by *Citrobacter ssp* or *Klebsiella ssp* that occurred in all of the untreated mice with influenza [72]. These data suggest that by inhibiting influenza virus infection as the primary cause of epithelial damage, DAS181 treatment may protect the airway epithelium from inflammation and denudation, which in turn may be the mechanism for prevention of secondary bacterial infection.

In Phase I clinical studies of DAS181 was well tolerated with no severe adverse events [73,74]. The safety and efficacy of DAS181 is now being further evaluated in Phase II clinical trials.

## 6. Combination Therapy

The potential advantage of using combination treatments against influenza virus was previously reviewed [26,75]. The combinations of antiviral treatments may offer additive or synergistic advantages over the respective monotherapy and could potentially reducing the development of drug-resistant influenza variants (for review [76]). Early clinical use of combination therapy using zanamivir and rimantadine [77], were showing trends toward decreased viral infection and reduced resistance of M2-inhibitor compared to groups treated with rimantadine alone. While, combinations of oseltamivir and ribavirin were effective against H5N1 in pre-clinical studies [78], ribavirin has some limitations due to toxicity. Most recently, combinations of NAIs and T-705 shown promise against H1N1 in mice [79], and may offer a safer compound to administer than ribavirin. Here, we examined the potential benefits of combination therapy of DAS181 and NAIs.

**Figure 2.**
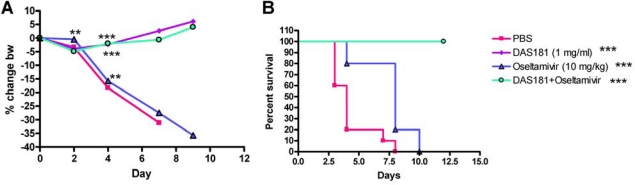
Combination with high infectious dose of Influenza A/PR/8/34. (A) % change body weight change. Differences were first analyzed by one way ANOVA of F-test for overall comparison among 6 groups. If statistically significant, followed by pair wise comparison (two sample t-test). At Day 7 there was only one animal left in PBS group, no pairwise comparison with PBS was made. At Day 9 there was no animal left in PBS group, no pairwise comparison with PBS was made. (B) survival curves during the course of infection and treatments. Mice (N = 10) were infected intranasally with 5,000 PFU of mouse-adapted A/PR/8/34 and 6 hours later received treatment (q.d. x5) with DAS181 (1mg/kg i.n), oseltamivir (10 mg/kg oral gavage), or a combination of the two drugs. Graphpad Prism 4.2 was used to assess statistical differences in survival curves. The statistical test was performed based on survival data in days. First log-rank test was used for overall comparison among 4 groups, if it is statistically significant, then pair wise comparison of log-rank test was performed. Asterisks indicate significant difference from vehicle treated groups as determined by Students T-Test ** = P < 0.01 *** = P < 0.001.

A number of animal studies have been conducted to investigate effects of DAS181 in combination with NAIs. In one study, mice were infected with a high infectious dose (>50-fold over the LD_100_) of the mouse-adapted influenza virus A/PR/8/34 (H1N1) strain and treated with DAS181 or oseltamivir alone, or in combination (Figure 2). Results demonstrated that while oseltamivir was only partially effective at 10 mg/kg/day qd x5 (mean days to death 7.6 compared to 4.3 for control), 1 mg/kg/day of DAS181, alone or together with oseltamivir, provided complete protection (100% survival, mean day to death >21 days) demonstrating the relative potency of DAS181 treatment and the absence of negative interactions between oseltamivir and DAS181. DAS181 may rescue clinical cases that fail to respond to oseltamivir.

**Figure 3.**
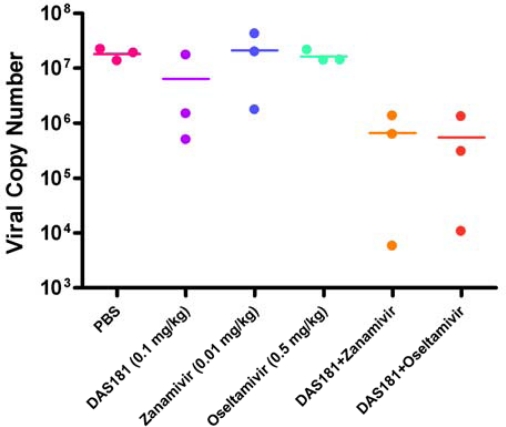
Combination of suboptimal treatment against lethal infection by A/PR/8/34. Mice (N = 10 per group) were infected intranasally (i.n) with 100 PFU of mouse-adapted A/PR/8/34. Treatment (q.d. x5) with DAS181 (0.1 mg/kg i.n), zanamivir (0.01 mg/kg i.n), oseltamivir (0.5 mg/kg oral gavage), alone or combination with DAS181 was initiated at 6 hr post-infection.

In another study, influenza virus infected mice were treated with DAS181, zanamivir, or oseltamivir, either alone or in combinations (Figure 3). The drug treatment doses were intentionally set at the sub-therapeutic dose levels to unmask potential synergistic drug-drug interactions. Results showed that while all the monotherapies failed to reduce viral replication, the combination treatments reduced the lung viral load on day 7 (Figure 3). Although the differences did not reach statistical significance due to the low number of replicates, these data suggested an additive or synergistic benefit of the combination treatments between NAIs and DAS181 in reducing viral replication.

## Conclusion

A number of novel drug candidates against influenza are currently under clinical development. Of particular importance for new influenza therapeutics is reduced risk of drug resistance. A host-targeting approach, represented by DAS181, may be particularly attractive due to a low drug-resistance potential as well as broad-spectrum activity. The merits of all therapeutic candidates against influenza will be revealed by future clinical trials.

## Figures and Tables

**Table 1.** New therapeutic candidates in development for influenza treatment.

Candidates	Mechanism of Action	Route of Delivery	Phase of Development	Developed By
Long-acting NAI (CS-8958)	Inhibition of viral release	Inhaled	Phase III (Japan) clinical trial	Biota/Daiichi-Sankyo
Peramivir	Inhibition of viral release	i.v	Phase III clinical trial*	BioCryst Pharmaceuticals
T-705 (favipiravir)	Inhibition of RNA replication	Oral	Phase II clinical trial	Toyama Chemical Co., Ltd (a subsidiary of Fuji Holdings)
DAS181 (Fludase®)	Inhibition of host cell receptor binding	Inhaled	Phase II clinical trial	NexBio Inc.

* Approved in Japan (2010). In 2009 FDA issued an Emergency Use Authorization for Peramivir, allowing the i.v. use of the drug for hospitalized patients in cases where other available treatment methods are ineffective or unavailable.
